# Association between Copy Number Variation and Response to Social Skills Training in Autism Spectrum Disorder

**DOI:** 10.1038/s41598-019-46396-1

**Published:** 2019-07-08

**Authors:** Kristiina Tammimies, Danyang Li, Ielyzaveta Rabkina, Sofia Stamouli, Martin Becker, Veronika Nicolaou, Steve Berggren, Christina Coco, Torbjörn Falkmer, Ulf Jonsson, Nora Choque-Olsson, Sven Bölte

**Affiliations:** 1 Center of Neurodevelopmental Disorders (KIND), Division of Neuropsychiatry, Centre for Psychiatry Research, Department of Women’s and Children’s Health, Karolinska Institutet, Solna, Sweden; 2 Child and Adolescent Psychiatry, Stockholm Health Services, Region, Stockholm, Sweden; 3 Curtin Autism Research Group, School of Occupational Therapy, Social Work and Speech Pathology, Curtin University, Bentley, Australia; 4 Pain and Rehabilitation Centre, Department of Medical and Health Sciences, Linköping University, Linköping, Sweden; 5 Department of Neuroscience, Child and Adolescent Psychiatry, Uppsala University, Uppsala, Sweden; 6 Center for Psychiatry Research, Department of Clinical Neuroscience, Karolinska Institutet, Stockholm, Sweden

**Keywords:** Genetics, Molecular medicine

## Abstract

Challenges in social communication and interaction are core features of autism spectrum disorder (ASD) for which social skills group training (SSGT) is a commonly used intervention. SSGT has shown modest and heterogeneous effects. One of the major genetic risk factors in ASD is rare copy number variation (CNV). However, limited information exists whether CNV profiles could be used to aid intervention decisions. Here, we analyzed the rare genic CNV carrier status for 207 children, of which 105 received SSGT and 102 standard care as part of a randomized clinical trial for SSGT. We found that being a carrier of rare genic CNV did not have an impact on the SSGT outcome measured by the parent-report Social Responsiveness Scale (SRS). However, when stratifying by pathogenicity and size of the CNVs, we identified that carriers of clinically significant and large genic CNVs (>500 kb) showed inferior SRS outcomes at post-intervention (*P* = 0.047 and *P* = 0.036, respectively) and follow-up (P = 0.008 and *P* = 0.072, respectively) when adjusting for *standard care* effects. Our study provides preliminary evidence that carriers of clinically significant and large genic CNVs might not benefit as much from SSGT as non-carriers. Our results indicate that genetic information might help guide the modifications of interventions in ASD.

## Introduction

Autism spectrum disorder (ASD) is a neurodevelopmental condition with a prevalence of 1–2%^1,2^. A multitude of alterations in social communication and interaction are among the core challenges in ASD^3^. The clinical presentation is heterogeneous, and the condition often co-occurs with other complications, such as attention-deficit hyperactivity disorder (ADHD), anxiety and depression, intellectual disability, and a variety of somatic disorders^4,5^. Reflecting the nature of the spectrum of clinical manifestations, the causes of ASD are diverse. Genetic factors, both common and rare play a significant part in the etiology of the disorder with complex interplay with each other and environmental factors^6,7^.

There is a scarcity of evidence-based interventions for ASD^8^. Social skills group training (SSGT) is a commonly used intervention to ameliorate social communication difficulties and their negative impact on adaptive outcomes in the normative intellectual range of ASD. SSGT is based on a combination of behavioral modification and socially instructive techniques applied in cohesive and safe peer group settings. The accumulated body of randomized controlled trials (RCTs) on SSGT suggest that the average effect is modest^9–11^. However, the overall heterogeneity of the outcomes indicates that subgroup analyses might help identify moderators and mediators. The largest RCT on SSGT to date evaluated the KONTAKT program^10^ led by our center, which compared the effect of *standard care plus SSGT* to *standard care alone*. The standard care consisted of any ongoing support or intervention provided by regular health care services (e.g., child and adolescent psychiatry, (neuro-)pediatrics, habilitation centers, speech, and language pathology services). Based on the primary outcome measure, parent-reported social responsiveness scale (SRS), the KONTAKT RCT did not show a significant overall effect of the 12-week training variant^10^. However, subgroup analyses showed that adolescents (13–17 years) improved significantly, but children (8–12 years) did not. In addition, females, rather than males, showed significant improvements^10^. Further, a previous uncontrolled trial of SSGT KONTAKT found language capacities to be positively correlated with outcome, while ASD severity negatively impacted the outcome^12^. Another previous SSGT trial did not find any association between ADHD, level of social anxiety, Theory-of-Mind, or desire for social interaction with the outcome^13^. However, the sample size was insufficient for informative subgroup analyses.

Genetic factors have not yet been studied as potential sources of heterogeneity in SSGT outcomes. The most convincing genetic risk markers in ASD are rare copy number variants (CNVs), which are deletions and duplications of genetic material. Rare CNVs confers up to a 20-fold increase risk for ASD^14^. The most frequent CNVs underlying ASD are 16p13.11 recurrent duplication/deletions, 15q11–q13 duplication (MIM: 608636), 1q21.1 deletion/duplication (MIM: 612474/612475), and 16p11.2 deletion (MIM:611913) syndromes^15^. Therefore, chromosomal microarray analysis (CMA) is recommended as the first-tier genetic test to identify CNVs in ASD with a molecular diagnostic yield up to 25%^16–18^. In addition to providing information about the genetic causes and recurrence risks for family members, CMA has been reported to result in individual medical intervention recommendations such as referral to specialists, medical follow-up and more detailed genetic investigations within the family^19,20^.

An unanswered question of high relevance for intervention planning in clinical practice is whether CMA results can guide the selection and prioritization of interventions in ASD at an individual or subgroup level. To address this question, we hypothesized that rare genic CNVs would have a potential moderating effect on the outcome following 12–weeks of SSGT KONTAKT. Our focus was especially on the rare genic CNVs, including chromosomal aneuploidies stratified further by clinical significance and size, to align our study with the current clinical recommendations of CMA evaluation in ASD.

## Results

### Sample characteristics

Data from the 12-week multicenter, randomized pragmatic clinical trial of the Swedish version of SSGT “KONTAKT” (hereafter referred to only as SSGT)^21^ and genetic screening were used for this study. Demographics and clinical characteristics of the participants included in this study are presented in Table 1, stratified by intervention group. Eighty-seven participants (29.3%) from the RCT were excluded from this study due to unavailable saliva sample for the genetic analysis. Of note, IQ was significantly lower in the SSGT participants without a saliva sample (Table S1). No other differences in the baseline measures were seen between the included and excluded participants (Table S1). The participants without saliva samples had a higher frequency of missing primary outcome data in both SSGT and standard care groups (Table S2). After genotyping and CNV quality control, two samples (one from SSGT and one from standard care group) were removed. Therefore, 105 children were included in the active SSGT group and 102 in the standard care group.

**Table 1:** Demographic and Clinical Characteristics at Baseline by Intervention Group.

Characteristics	SSGT (n = 105)	Standard care (n = 102)
Sex		
Female, n (%)	28 (26.7%)	32 (31.4%)
Male, n (%)	77 (73.3%)	70 (68.6%)
Age (mean, SD)	11.92 (±2.59)	11.48 (±2.69)
Adolescents, n (%)	39 (37.1%)	37 (36.3%)
Children, n (%)	66 (62.9%)	65 (63.7%)
Comorbidity		
ADHD, n (%)	77 (73.3%)	75 (73.5%)
Anxiety, n (%)	23 (21.9%)	23 (22.5%)
Depression, n (%)	13 (12.4%)	16 (15.7%)
Others, n (%)	16 (15.2%)	14 (13.7%)
1 Comorbidity, n (%)	83 (79.0%)	77 (75.5%)
2 or more comorbidities, n (%)	22 (21.0%)	25 (24.5%)
Cognitive and Behavioral Measures		
IQ (mean, SD)	98.48 (±13.08)	98.43 (±12.94)
SRS pre-intervention (mean, SD)	86.08 (±25.06)	86.33 (±24.30)
ADOS total score (mean, SD)	10.60 (±3.31)	10.95 (±3.45)
DD-CGAS (mean, SD)	58.26 (±7.27)	57.13 (±7.02)
OSU Autism CGI-S (mean, SD)	4.23 (±0.83)	4.41 (±0.79)
ABAS-II (mean, SD)	371.95 (±73.0)	374.66 (±61.89)

Abbreviations: SSGT, Social Skills Group Training; ADHD, attention-deficit hyperactivity disorder; SRS, Social Responsiveness Scale; ADOS, the Autism Diagnostic Observation Schedule; DD-CGAS, Developmental Disabilities modification of the Children’s Global Assessment Scale; OSU Autism CGI-S, Ohio State University (OSU) Global Severity Scale for Autism; ABAS-II the Adaptive Behavior Assessment System II.

### Characteristics of rare genic CNV carriers

Of the 207 included participants, 71 (34.8%) carried at least one rare genic CNV ≥25 kb (Table S3). Of these, 14 (6.8%) were carriers of large CNVs (including the chromosomal aneuploidies), 42 (20.3%) carriers of middle-size CNVs, and 15 (7.2%) carriers of small CNVs. We additionally categorized the CNVs based on their clinical significance using the recommended guidelines^22^. In total, 17 individuals (8.2%) had one or two clinically significant CNVs (pathogenic or likely pathogenic CNVs). Of these, three participants (1.45%) were carriers of sex chromosome aneuploidies (47, XXX; 45, X; 47; XYY). Additionally, we found large deletions and duplications affecting known risk CNV loci (15q11.2–q13.1; 9p24.3–p23; 16p13.11, 7q11.2, Xq27.3–Xq27.2), and smaller CNVs affecting genes with strong evidence to be involved in ASD or related conditions such as *GATAD2B* (OMIM*614998), *CHD8* (OMIM*610528), *ASH1L* (OMIM*607999), *KDM6A* (OMIM*300128), and *KDM5B* (OMIM*605393). We performed experimental validation using quantitative polymerase chain reaction (qPCR) for 18% of the identified rare variants with a 100% validation rate.

The rare genic and clinically significant CNV carriers in the SSGT group had significantly lower average IQ in comparison with the non-carriers (T = 2.03, *P* = 0.045 and T = 2.44, *P* = 0.016, respectively) but not in the whole sample (T = 1.69, p = 0.091, T = 1.40, *P* = 0.16) (Fig. S1). Additionally, Autism Diagnostic Observation Schedule (ADOS) total scores were higher in the carriers of rare genic CNVs >500 kb in the total sample (T = 2.05, *P* = 0.041). No other significant differences at baseline were observed for the primary outcome measure SRS total raw scores or ADOS scores between the CNV carriers and non-carriers (Fig. S1).

### Association between intervention response and carrier status of rare genic CNVs

**Figure 1:**
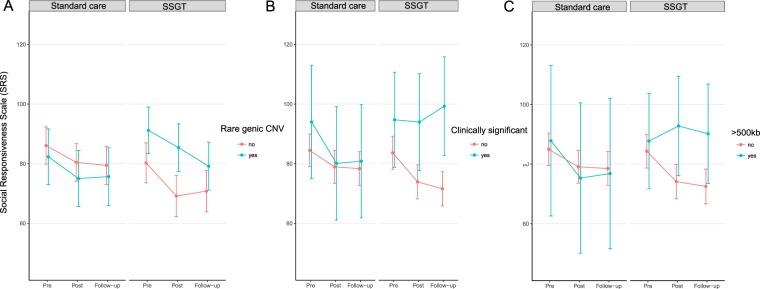
Least-square means and 95% confidence interval for mixed-linear model for parent-rated Social Responsiveness Scale (SRS) scores for the standard care and SSGT groups stratified to carriers of rare genic copy number variation (CNV) and non-carriers (**A**), carriers and non-carriers of clinically significant CNVs (**B**) and carriers and non-carriers of CNVs; >500 kb and chromosomal aneuploidies (**C**).

We aimed to test the hypothesis that the carrier status of rare genic CNVs or CNV subgroups, based on the pathogenicity or size, would be associated with the SSGT outcome. To test the associations, we performed the following comparisons: a) carriers of rare genic CNVs in comparison with non-carriers followed with subgroup analyses, b) carriers of clinically significant CNVs in comparison with all the participants without these CNVs and, c) CNV size stratified group comparisons. The results of the mixed linear models (MLM) adjusted for sex and age group (children or adolescents) for the primary outcome measure SRS are reported in Table 2, and the estimated Least Square (LS)-means are shown in Fig. 1. No differences were found for the carriers of rare genic CNVs at post-intervention or follow-up in comparison with non-carriers.

**Table 2:** Model Estimates of Associations Between Rare Copy Number Variation and Primary Outcome.

Mixed Linear Model	SSGT (n = 105)		Total Sample (N = 207)	
	β^a^ (95% CI)	P value	β^a^ (95% CI)	P value
Carrier of a rare CNV				
*SRSpost	5.27 (−2.1, 12.6)	0.16	−1.66 (−9.0, 5.7)	0.66
*SRSfu	−2.59 (−10.0, 4.9)	0.50	−0.02 (−7.8, 7.8)	1.0
*SRSpost*SSGT	—	—	6.99 (−3.1–17.1)	0.18
*SRSfu*SSGT	—	—	−2.52 (−13.0, 8.0)	0.64
Carrier of a clinically significant CNV				
*SRSpost	9.12 (−2.83, 21.07)	0.137	−4.25 (−9.20, 0.71)	0.094
*SRSfu	16.59 (4.14, 29.05)	**0.010**	−5.96 (−11.10, −0.83)	**0.023**
*SRSpost*SSGT	—	—	17.34 (0.30, 34.37)	**0.047**
*SRSfu*SSGT	—	—	23.64 (6.24, 41.04)	**0.008**
Carrier of a rare large CNV (>500 kb)				
*SRSpost	15.35 (2.9, 27.8)	**0.017**	−6.7 (−23.3. 10.0)	0.43
*SRSfu	14.19 (1.7, 26.7)	**0.028**	−4.57 (−21.24, 12.1)	0.59
*SRSpost*SSGT	—	—	21.90 (1.5, 42.3)	**0.036**
*SRSfu*SSGT	—	—	18.82 (−1.6, 39.3)	0.072

Abbreviations: SRSpost, Social Responsiveness scale outcome at post-intervention; SRSfu, Social Responsiveness Scale outcome at follow-up; SSGT, Social skills group training; CI, confidence interval. “–” indicates not calculated. ^a^Negative β-estimate indicates decrease (symptoms decrease) and positive estimate increase (symptoms increase) of the SRS score from pre-intervention to post-intervention and follow-up.

In our subgroup analyses, the carriers of clinically significant CNVs showed poorer outcome than participants without these CNVs at follow-up (β = 16.59, 95% CI 4.14–29.05, *P* = 0.010) (Table 2, Fig. 1). Levels of statistical significance increased when including the ‘standard care’ group in the three-way interaction model, both at post-treatment (β = 17.34, 95% CI 0.30–34.37, *P* = 0.047) and at follow-up (β = 23.64 95% CI, 6.24–41.04, *P* = 0.008) (Fig. 1, Table 2). Changes between pre- and post-intervention/follow-up for the carriers of clinically significant CNVs and non-carriers are shown in Fig. S2. As we observed IQ differences between the CNV-carriers and non-carriers in the SSGT group, IQ–adjusted models were computed. The association seen for the clinically significant CNVs in the SSGT group was not significant after adjustment. However,the three-way interaction model remained unchanged (Table S5).

In the size stratification approach, the carriers of large genic CNVs (>500 kb), including the chromosomal aneuploidies, also had inferior outcome at post-intervention (β = 15.35, 95% CI 2.9–27.8, *P* = 0.017) and at follow-up (β = 14.19, 95% CI 1.7–26.7, *P* = 0.028) compared with participants without large genic CNVs (Table 2, Fig. 1, Figure S2). In the three-way interaction model, the significant effect for large genic CNV carriers in SSGT group remained at post-intervention (β = 21.90, 95% CI 1.5–42.3, *P* = 0.036) with a similar trend at follow-up (β = 18.82, 95% CI −1.6–39.3, *P* = 0.072) (Table 2, Fig. 1). Putative positive association was observed for carriers of middle-size CNVs at follow-up (β = −8.69, 95% CI −16.8–0.6, *P* = 0.038) in the SSGT group; however, the comparison did not reach significance when including the ‘standard care group’ to the model. The models adjusted for IQ were unchanged (Table S5).

### Association between secondary outcomes and rare genic CNV carrier status

The results for the secondary outcomes, the parent-rated Adaptive Behavior Assessment System II (ABAS-II), trainer-rated Developmental Disabilities modification of the Children’s Global Assessment Scale (DD-CGAS) and the Ohio State University (OSU) Global Severity Scale for Autism (OSU Autism CGI-S) are presented in Table S6 with IQ-adjusted results presented in Table S7. The three-way interaction model for time and intervention effects for ABAS-II parent rating showed a decrease in adaptive functioning for the carriers of clinically significant CNV at post-intervention and follow-up (β = −39.82, 95% CI −76.67– −2.98, *P* = 0.035 and β = −43.02, 95% CI−80.67– −5.37, *P* = 0.026, respectively). The carriers of large rare genic CNVs also showed decreased adaptive functioning as measured by ABAS-II at post-intervention (β = −28.75, 95% CI −56.6–0.9 *P* = 0.045); however, the decreased adaptive functioning did not remain significant in the three-way interaction model (β = −40.39, 95% CI −84.5–3.7 *P* = 0.073). For the trainer-ratings using OSU Autism CGI-S, carriers of small CNVs showed greater improvements at post-intervention (β = −1.20, 95% CI −2.0–0.43 *P* = 0.003) with significant three-way interaction model at same time point (β = −1.21, 95% CI −2.1–0.3 *P* = 0.008). No other significant comparisons were found for the SSGT or three-way interaction MLMs (Table S6). The IQ-adjusted model showed similar results (Table S7).

## Discussion

This is the first study to analyze the moderating effects of rare genic CNVs on SSGT intervention in children and adolescents with ASD. Our results suggest that, in general, being a carrier of any size rare genic CNV did not have an impact on the primary outcome. However, subgroup analyses showed that carriers of clinically significant and large genic CNVs (>500 kb), including chromosome aneuploidies, improved less than non-carriers in the 12-week SSGT training as reported by the parents. Previous studies have reported that many of the recurrent large rare CNVs are associated with low IQ^23,24^. We demonstrate that the carriers of rare genic or clinically significant CNVs had lower IQ than non-carriers in the SSGT group, but not in the total sample, showing an inconsistent pattern. Despite the differences, the IQ-adjusted main results in the total sample remained unchanged, indicating effects of the CNVs beyond IQ. The inferior outcomes of carriers of clinically significant and large rare genic CNVs were robust when analyzing parent-rated outcome measures but were not replicated in trainer-rated secondary outcomes. Our results suggest that genetic information might help to guide the modifications of intervention programs for certain ASD subgroups, as well as the choice of treatment to achieve better personalization of intervention planning and clinical resource allocation.

Hopefully, the recent molecular evidence in ASD can be used to design effective medical interventions targeting ASD core symptoms^25^. However, there is still a long road ahead until such interventions can be introduced on the market and implemented in clinical practice. Therefore, it is crucial to investigate how genetic information can help enhance behavioral treatment decisions and modifications using current and widely applied ASD programs. Studies on potential genetic moderators of both pharmacological and behavioral treatments in psychiatry have begun to emerge in recent years^26,27^. For instance, a recent study showed that individuals with bipolar disorder and high genetic load as measured by polygenic scores for schizophrenia had an inadequate response to lithium treatment^28^. Additionally, a study investigating the association of CNVs with the response to antidepressant medication in major depressive disorder showed no general effect, but the authors noted less response for carriers of CNVs in specific loci, including *NRXN1* (OMIM* 600565)^29^. For genetic moderators of behavioral interventions, recent studies have mostly used common genetic variants either in selected candidate genes^30^ or a hypothesis-free genome-wide approach^31^ with no or limited evidence of genetic moderators of treatment response. However, recently polygenic risk score for ASD was shown to modulate the response to cognitive behavior therapy in patients with major depression^32^. Here, we used a different approach, by investigating rare genic CNVs that are associated with a large risk for ASD as well as other neurodevelopmental and psychiatric disorders^33,34^. To the best of our knowledge, our study is the first to investigate genetic moderators of behavioral intervention outcome in ASD.

Our data also show that CMA is useful in testing for genetic alterations in autistic individuals in the average and high intellectual range. We identified rates of clinically significant CNVs (8.2%) similar to what has previously been reported in samples, including individuals with ASD in the intellectual disability range^16–18^. Interestingly, our results seem to corroborate previous studies showing that individuals with ASD and genomic or monogenic syndromes form a subgroup characterized by lower cognitive abilities and milder ASD symptomatology, especially for social communication alterations^35,36^. Similarly, an earlier study using machine learning classification showed that individuals with specified genetic disorders in ASD have a specific signature of social impairment^37^. Interestingly, in our study the carriers of clinically significant CNVs showed better improvements than the non-carriers in the standard care group (Fig. 1), which was also indicated by all the secondary outcome measures (Table S6). Therefore, it is crucial to better understand the intervention needs of those with specified molecular alterations. For instance, it should not be ruled out that this group could benefit from longer periods of training. We recently reported that a long version of KONTAKT (24-weeks) resulted in larger effects in general than we have found for the 12-week version used here^38^. Unfortunately, that trial was not sufficiently powered for subgroup analyses.

Limitations of our study should be noted. First, while this study was based on the largest RCT in ASD research to date, the sample was still limited to investigate the effects of single loci. Thus, our results reflect various molecular causes. Second, data on either saliva or the primary outcome were missing for 87 (29%) of the participants enrolled in the trial, and these missing participants from the active SSGT group had significantly lower IQ and missing outcome measure data. Putatively, these participants could have a higher frequency of the clinically significant CNVs as described in the literature^23,24^. Third, the secondary outcome measures provided a somewhat inconsistent picture, underscoring the preliminary nature of the results. Fourth, the significant subgroup differences should be interpreted with caution. These limitations must be addressed in future studies before specific clinical recommendations can be made based on CNVs. From a clinical and practical perspective, we would also like to stress that there are many social, economic, legal, and ethical issues to be addressed as the genetic and genomic testing in ASD is rapidly increasing. These include, but are not limited to, realistic cost-benefit analyses, genetic data protection, clinical guideline development, and the avoidance of discrimination of genetic and ethnic minorities^6,39,40^.

## Conclusions

This is the first study to show that individuals with ASD who have clinically significant or large rare genic CNVs are less likely than non-carriers to benefit from SSGT and might need more specifically modified and tailored SSGT or other behavioral intervention programs. Until replicated in independent samples, our results should be interpreted with caution.

## Methods

### Study design

The initial 12-week multicenter, randomized pragmatic clinical trial of the Swedish version of SSGT “KONTAKT”^21^ tested the effectiveness of SSGT as a complement to standard care in 13 child and adolescent psychiatry outpatient units in Sweden. Outcome measures were collected at pre-, post-intervention (12-weeks), and follow-up (3-months after post-intervention). The protocol and results from the trial have been published previously^10,21^. The study protocol and all the methods have been performed in accordance with the Declaration of Helsinki and approved by the ethical review board in Stockholm, Sweden (Dnr 2012/385-31/4) and the clinical authorities of the two involved counties. The trial was also registered online (NCT01854346). Written informed consent from the parents or legal guardians and verbal consent from the children and youth were collected. The trial and the collection of saliva samples were done between August 2012 and October 2015.

### Participants

The original trial included 296 youths aged 8 to 17 years with ASD. Eligibility criteria and recruitment of participants are described in detail elsewhere^10^. In short, participants had a clinical consensus diagnosis of autism, atypical autism, Asperger syndrome, or pervasive developmental disorder not otherwise specified based on ICD-10 criteria^41^. The diagnosis was corroborated by ASD cutoffs (modules 3 or 4) on the ADOS^42^. Additionally, the ADOS total scores were used to estimate the autism severity at baseline. All participants had full-scale IQs >70, according to the Wechsler Intelligence Scale for Children (third or fourth edition)^43^ and at least one common comorbid psychiatric ICD-10 diagnosis of ADHD, anxiety disorder, or mood disorder. Participants providing saliva samples and primary outcome data for at least one-time point (post-intervention or 3-month follow-up) were included in this study to investigate the role of CNV carrier status in the SSGT outcome (N = 209; 106 from the SSGT group, 103 from the ‘standard care’ group). We also used the baseline measures from the participants without saliva samples (n = 87) to analyze if any differences were observed between the included and excluded participants.

### Outcome measures

The primary outcome measure was the change in total raw scores on the parent reported SRS^44^. Higher values on the SRS scale indicate greater severity of autistic symptoms. Secondary outcome measures used in the RCT were parent ratings on the ABAS-II^45^, the trainer-rated DD-CGAS^46^, and OSU Autism- CGI-S^47^.

### Genotyping and CNV calling

The participants collected saliva samples using the Oragene•DNA OG-500 tubes (DNA Genotek, Inc., Ottawa, Ontario, Canada) at home using a recommended procedure. Thereafter, the DNA was extracted using Chemagen kit (PerkinElmer chemagen, Baesweiler, Germany) with chemagicSTAR®-robot (Hamilton Robotics, Reno, NV, USA). The genotyping of the DNA samples were done on the Affymetrix CytoScan™ HD microarray platform (Santa Clara, CA, USA), which has approximately 2.7 million probes, following the manufacturer’s recommendations. The identification of CNVs was made by incorporating calls from two algorithms Chromosome Analysis Suite (ChAS) software v.3.1 (Affymetrix), and Partek Genomics Suite software, version 6.6 (Partek Inc., St.Louis, MO, USA). Standard quality control was performed for the single nucleotide polymorphism (SNP) and CNV data.

For the statistical analyses, we considered only variants that were called by both algorithms and spanned at least 25 kb and 25 consecutive probes^48^. If large CNVs were found to affect the sex chromosomes, and SNP data showed inconsistent information of gender of the participants, the calls were manually inspected using ChAS software (Affymetrix) to verify the call and check for possible chromosomal aneuploidies. As we sought to limit analyses to CNVs that would be included in clinical CMA reports, we removed variants that were present with more than 0.1% frequency in the general population using the Ontario Population Genomics Platform (OPGP) data as a reference^48^ In OPGP, 873 samples were genotyped using the same Affymetrix platform. Additionally, we removed variants that overlapped with more than five variants (50% reciprocal length) that are reported in the Database of Genomic Variants (DGV)^49^. The CNVs were mapped using GRCh37/hg19 coordinates. Thereafter, CNVs that overlapped at least one coding exon based on the gene annotation from RefSeq were included in the final CNV set. The rare genic CNVs were categorized based on their pathogenicity as recommend by the American College of Medical Genetics and Genomics^22^, and by size into three groups: 25–100 kb (small), 101–500 kb (middle-size), and >500 kb including chromosome aneuploidies (large), an approach used in earlier literature^14,50^. We performed experimental validation of 15 selected CNVs using qPCR.

### Statistical analyses

CNV status was coded binary with “0” for non-carriers and “1” for carriers. The rare genic CNVs were categorized into clinically significant CNVs and by size into three size groups. The participants were grouped according to the largest CNV present. The effects of all rare genic CNVs, clinically significant, and the three CNV size groups, on the primary and secondary outcome measures, were tested separately (as they represent overlapping groups) using mixed linear models (MLM). Based on our primary hypothesis that rare CNVs would affect the outcome of SSGT, we first tested the two-way interaction of CNV carrier*time (post-intervention and 3-month follow-up) in the active SSGT participant group. Thereafter, we examined the models adding the standard care group in a three-way interaction CNV carrier*time*intervention group (SSGT vs. standard care). Our primary MLM included the fixed effects of age group (children or adolescents) and sex, as these had shown to influence intervention outcome in the KONTAKT SSGT RCT study^10^. To account for the putative differences between participating centers and individuals, these were included as random effects in the models. The least-square (LS) means, and 95% confidence interval (CI) were calculated. The adjusted models included IQ as a covariate based on previous studies, suggesting an association of CNVs with IQ and educational attainment in the general population^23,24^. Additionally, we tested for differences in clinical group characteristics at baseline (SRS, IQ, ADOS total score) between the participants included and excluded in this study within the SSGT and standard care group as well as between rare genic CNV carriers and non-carriers using two-sided student’s t-test and χ^2^ test. The analyses were conducted using R version 3.4.2.

## Supplementary information

Supplement

Dataset 1

## Data Availability

The raw array data or phenotypic data has not been shared in a public database due to the limited ethical approval, and informed consent from the participants for data sharing but are available from the corresponding author (kristiina.tammimies@ki.se) upon request and subject to necessary clearances.
