# Supplementary material for: Association between Copy Number Variation and Response to Social Skills Training in Autism Spectrum Disorder
Source: Sci Rep. 2019 Jul 8;9:9810. doi: 10.1038/s41598-019-46396-1 (PMC6614458; doi:10.1038/s41598-019-46396-1)
Supplement: Supplementary file 1 — Supplement [file 41598_2019_46396_MOESM1_ESM.pdf]

Supplementary Material

Association between Copy Number Variation and Response to Social Skills Training in Autism Spectrum Disorder

Tammimies K, Li D, Rabkina I et al.

Contents

Figure S1. Box-plots showing the baseline measures between the carriers of A) Rare genic copy number variation (CNV), B) Clinically significant CNV and C) Rare genic CNV >500 kb and non-carriers in the active social skills training group (SSGT) (n=105) and the Total Sample (N=207). 2

Figure S2. Box-plots showing the distribution of the changes in Social Responsiveness Scale (SRS) in carriers of rare genic copy number variants (CNVs) (yes) and non-carriers (no) at post-intervention (SRS pre – SRS post-intervention) and follow-up (SRS pre – SRS follow-up) (A) and stratified by pathogenicity (B) or by size of the CNV (C-E). 3

Table S1. Comparison Between the Baseline Measures of Included and Excluded Participants within the Social Skills Group Training (SSGT) and 'Standard Care' 4

Table S2. Comparison Between the Complete Primary Outcome Data of Included and Excluded Participants Within the Social Skills Group Training (SSGT) and 'Standard Care' 5

Table S3. List of rare genic CNVs found in the study individuals (additional file) 6

Table S4. Mixed linear model results for the association between rare genic copy number variation (CNV) and the primary outcome measure social responsiveness scale (SRS) in the active social skills group training (SSGT) participants and the total sample for carriers of middle-size (101-500 kb) and small rare genic CNVs (25-100 kb). 6

Table S5. IQ adjusted mixed linear model results for the association between rare genic copy number variation (CNV) and the primary outcome measure social responsiveness scale (SRS) in the active social skills group training (SSGT) participants and the total sample. 7

Table S6. Mixed linear model results for the association between copy number variation (CNV) and the secondary outcome measures in the active social skills group training (SSGT) participants and the total sample. 8

Table S7. IQ adjusted mixed linear model results for the association between rare genic copy number variation (CNV) and the secondary outcome measures in the active social skills group training (SSGT) participants and the total sample 10

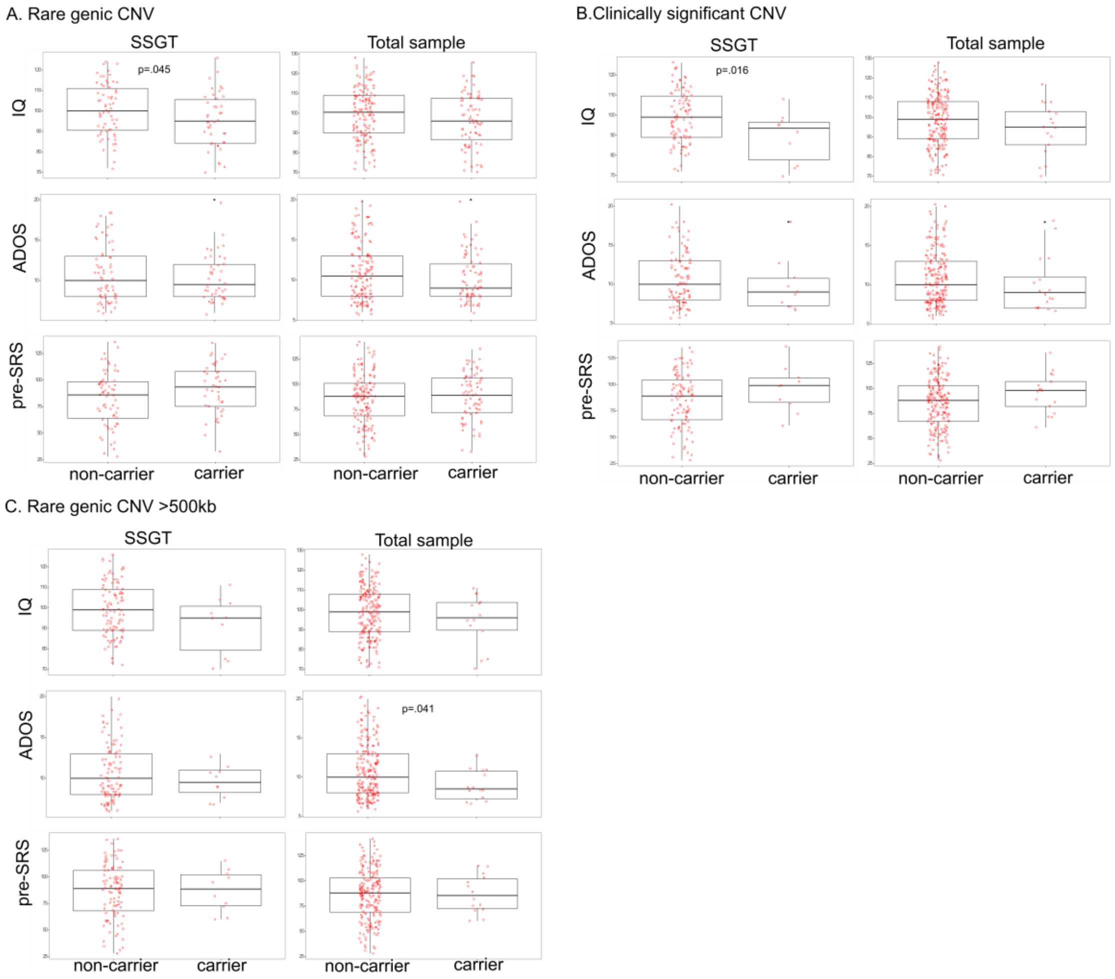

Figure S1. Box-plots showing the baseline measures between the carriers of A) Rare genic copy number variation (CNV), B) Clinically significant CNV and C) Rare genic CNV >500 kb and non-carriers in the active social skills training group (SSGT) (n=105) and the Total Sample (N=207). Social Responsiveness Scale (SRS), Full-Scale IQ, and the Autism Diagnostic Observation Schedule (ADOS) Total Score. *P* values for two-sided Student's T-tests are shown for the significant comparisons.

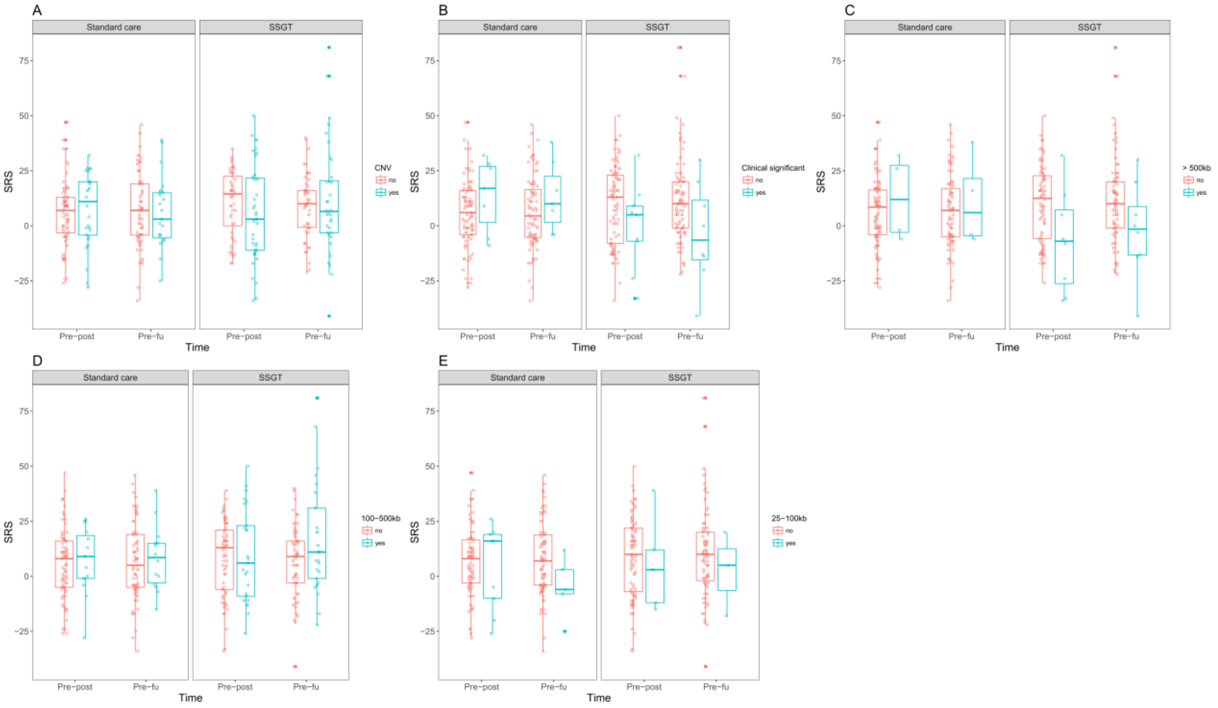

Figure S2. Box-plots showing the distribution of the changes in Social Responsiveness Scale (SRS) in carriers of rare genic copy number variants (CNVs) (yes) and non-carriers (no) at post-intervention (SRS pre – SRS post-intervention) and follow-up (SRS pre – SRS follow-up) (A) and stratified by pathogenicity (B) or by size of the CNV (C-E).

Table S1. Comparison Between the Baseline Measures of Included and Excluded Participants within the Social Skills Group Training (SSGT) and 'Standard Care'

	**SSGT**		
	**Excluded participants due to unavailable saliva sample (n = 44)**	**Included participants (n = 105)**	***P* value[a]**
Full Scale IQ mean (SD)	93.38 (13.78)	98.49 (13.08)	**.038**
ADOS total score	10.76 (4.05)	10.60 (3.31)	.806
SRS pre-intervention	92.13 (21.81)	86.08 (25.06)	.140
	**Standard care**		
	**Excluded participants due to unavailable saliva sample (n = 43)**	**Included participants (n = 102)**	***P* value[a]**
IQ mean (SD)	97.43 (12.96)	98.43 (12.94)	.670
ADOS total score	11.20 (4.22)	10.95 (3.28)	.704
SRS pre-intervention	91.41 (26.97)	86.33 (24.30)	.286

Abbreviations: ADOS, Autism Diagnostic Observation Schedule; SRS, Social Responsiveness Scale. [a] statistical significance was computed using two-tailed Student T-test.

Table S2. Comparison Between the Complete Primary Outcome Data of Included and Excluded Participants Within the Social Skills Group Training (SSGT) and 'Standard Care'

**SSGT**			
	Excluded participants due to missing saliva sample (n = 44)	Included participants (n = 105)	***P* value**[a]
**Missing SRS at post-intervention**	18	15	**<.001**
**Post-intervention SRS**	26	90	
**Missing follow-up SRS**	24	19	**<.001**
**Follow-up SRS**	20	86	
**Standard care**			
	Excluded participants due to missing saliva sample (n = 43)	Included participants (n = 102)	***P* value**[a]
**Missing SRS at post-intervention**	11	6	**.002**
**Post-intervention SRS**	32	96	
**Missing follow-up SRS**	22	19	**<.001**
**Follow-up SRS**	21	83	

Abbreviations: SRS, Social Responsiveness Scale. [a]Statistical significance was computed using a two-sided $\chi 2$ test.

Table S3. List of rare genic CNVs found in the study individuals (additional file)

Table S4. Mixed linear model results for the association between rare genic copy number variation (CNV) and the primary outcome measure social responsiveness scale (SRS) in the active social skills group training (SSGT) participants and the total sample for carriers of middle-size (101-500 kb) and small rare genic CNVs (25-100 kb).

	**SSGT (n=105)**		**Total Sample (N=207)**	
**Mixed Linear Model**	**β (95% CI)[$]**	**P value**	**β (95% CI)**	**P value**
Carrier of middle size CNV (101-500 kb)				
*SRSpost	-0.77 (-8.8, 7.3)	.85	-2.05 (-11.2, 7.1)	.66
*SRSfu	-8.69 (-16.8, -0.6)	**.038**	-3.46 (-12.9, 6.0)	.47
*SRSpost*SSGT	.	-	1.32 (-10.6, 13.2)	.83
*SRSfu*SSGT	-	-	-5.25 (-17.4, 6.9)	.40
Carrier of small CNV (25-100 kb)				
*SRSpost	2.93 (-13.1, 18.9)	.72	2.32 (-9.2, 13.8)	.69
*SRSfu	- 0.78 (-20.1, 18.6)	.94	11.00 (-3.2, 25.2)	.13
*SRSpost*SSGT	-	-	0.93 (-17.9, 19.8)	.92
*SRSfu*SSGT	-	-	-11.58 (-34.6, 11.4)	.33

[$]Negative β estimate indicates decrease (symptoms decrease) and positive estimate increase (symptoms increase) of the SRS score from pre-intervention to post-intervention and follow-up for the carriers.

Table S5. IQ adjusted mixed linear model results for the association between rare genic copy number variation (CNV) and the primary outcome measure social responsiveness scale (SRS) in the active social skills group training (SSGT) participants and the total sample.

	SSGT KONTAKT group			Total sample		
**Social Responsiveness Scale (SRS)**	**β$**	95% CI	*P*	**β$**	95% CI	*P*
**Carrier of a rare genic CNV**						
*SRSpost	5.33	-2.00, 12.65	.16	-1.73	-9.06, 5.60	.64
*SRSfu	-2.53	-9.97, 4.94	.51	-0.03	-7.86, 7.80	.99
*SRSpost*SSGT	-	-	-	7.09	-2.99, 17.17	.17
*SRSfu*SSGT	-	-	-	-2.44	-12.97, 8.09	.65
**Carrier of a clinically significant CNV**						
*SRSpost	1.29	-7.22, 9.80	.77	-4.20	-9.15, 0.75	.097
*SRSfu	5.60	-3.13, 14.32	.21	-6.03	-11.16, -0.90	**.023**
*SRSpost*SSGT	-	-	-	17.28	0.24, 34.31	**.047**
*SRSfu*SSGT	-	-	-	23.51	6.11, 40.91	**.008**
**Carrier of large size CNV (>500 kb)**						
*SRSpost	15.08	2.60, 27.56	**.019**	-6.616	-23.247, 10.015	.44
*SRSfu	13.86	1.36, 26.36	**.031**	-4.608	-21.281, 12.064	.59
*SRSpost*SSGT	-	-	-	21.676	1.269, 42.082	**.038**
*SRSfu*SSGT	-	-	-	18.693	-1.758, 39.144	.074
**Carrier of middle size CNV (101-500 kb)**						
*SRSpost	-0.74	-8.82, 7.33	.86	-2.19	-11.33, 6.96	.64
*SRSfu	-8.70	-16.83, -0.57	**.038**	-3.60	-13.03, 5.84	.46
*SRSpost*SSGT	-	-	-	1.50	-10.41, 13.41	.81
*SRSfu*SSGT	-	-	-	-5.09	-17.25, 7.08	.41
**Carrier of small CNV (20-100 kb)**						
*SRSpost	3.45	-12.53, 19.43	.67	2.35	-9.14, 13.85	.69
*SRSfu	0.19	-19.12, 19.49	.99	11.06	-3.15, 25.26	.13
*SRSpost*SSGT	-	-	-	1.14	-17.74, 20.01	.91
*SRSfu*SSGT	-	-	-	-10.99	-33.99, 12.02	.35

Abbreviations: SRSpost, Social Responsiveness Scale outcome at post-intervention; SRSfu, Social Responsiveness scale outcome at follow-up. "–" indicates not calculated.
$Negative β estimate indicates decrease (symptoms decrease) and positive estimate increase (symptoms increase) of the SRS score from pre-intervention to post-intervention and follow-up for the carriers.

Table S6. Mixed linear model results for the association between copy number variation (CNV) and the secondary outcome measures in the active social skills group training (SSGT) participants and the total sample.

	**SSGT KONTAKT group**			**Total sample**		
**ABAS-II (parent-rated)**	**β$**	95%CI	*P*	**β$**	95%CI	*P*
**Carrier of a rare CNV**						
*ABAS-II_post	10.83	-5.37, 27.02	.19	-6.39	-22.223, 9.438	.43
*ABAS-II_fu	9.36	-7.22, 25.95	.27	4.15	-12.78, 21.09	.63
*ABAS-II_post*SSGT	-	-	-	17.25	-4.44, 38.95	.12
*ABAS-II_fu*SSGT	-	-	-	5.00	-17.75, 27.74	.67
**Carrier of a clinically significant CNV**						
*ABAS-II_post	-22.45	-49.17, 4.26	.10	15.94	5.29, 26.59	**.0036**
*ABAS-II_fu	-14.65	-42.52, 13.23	.30	14.19	3.07, 25.31	**.013**
*ABAS-II_post*SSGT				-39.82	-76.67, -2.98	**.035**
*ABAS-II_fu*SSGT				-43.02	-80.67, -5.37	**.026**
**Carrier of a rare large CNV (>500 kb)**						
*ABAS-II_post	-28.75	-56.63, -0.88	**.045**	12.16	-23.78, 48.10	.51
*ABAS-II_fu	-8.72	-36.66, 19.27	.54	29.06	-6.97, 65.10	.12
*ABAS-II_post*SSGT	-	-	-	-40.39	-84.48, 3.70	.073
*ABAS-II_fu*SSGT	-	-	-	-37.78	-81.99, 6.42	.095
**Carrier of middle size CNV (101-500 kb)**						
*ABAS-II_post	17.80	0.07, 35.54	.051	-7.58	-27.36, 12.19	.45
*ABAS-II_fu	9.48	-8.59, 27.55	.31	-4.77	-25.18, 15.64	.65
*ABAS-II_post*SSGT	-	-	-	25.22	-0.40, 50.83	.054
*ABAS-II_fu*SSGT	-	-	-	14.23	-12.07, 40.53	.29
**Carrier of small CNV (25-100 kb)**						
*ABAS-II_post	28.96	-9.22, 67.14	.14	-9.26	-34.01, 15.50	.46
*ABAS-II_fu	24.86	-17.92, 67.64	.26	4.19	-26.45, 34.84	.79
*ABAS-II_post*SSGT	-	-	-	38.28	-4.61, 81.17	.081
*ABAS-II_fu*SSGT	-	-	-	19.63	-30.16, 69.428	.44
**OSU Autism CGI-S (trainer-rated)**	**β#**	95%CI	*P*	**β#**	95%CI	*P*
**Carrier of a rare CNV**						
*CGI-S_post	-0.01	-0.35, 0.33	1.0	0.07	-0.26, 0.40	.68
* CGI-S_fu	-0.11	-0.45, 0.23	.53	-0.05	-0.39, 0.29	.78
* CGI-S_post*SSGT	-	-	-	-0.08	-0.53, 0.37	.72
* CGI-S_fu*SSGT	-	-	-	-0.05	-0.51, 0.41	.82
**Carrier of a clinically significant CNV**						
*CGI-S_post	0.11	-0.45, 0.67	.71	-0.35	-0.58, -0.13	**.0020**
* CGI-S_fu	-0.045	-0.62, 0.54	.88	-0.38	-0.61, -0.15	**.0011**
* CGI-S_post*SSGT				-0.03	-0.80, 0.74	0.94
* CGI-S_fu*SSGT				0.14	-0.66, 0.95	0.73
**Carrier of a rare large CNV (>500 kb)**						
* CGI-S_post	-0.07	-0.63, 0.49	.81	0.12	-0.63, 0.87	.75
* CGI-S_fu	-0.23	-0.83, 0.38	.46	-0.48	-1.31, 0.35	.26
* CGI-S_post*SSGT	-	-	-	-0.20	-1.11, 0.71	.67
* CGI-S_fu*SSGT	-	-	-	0.24	-0.75, 1.23	.64
**Carrier of middle size CNV (101-500 kb)**						

* CGI-S_post	0.29	-0.08, 0.66	.13	0.07	-0.34, 0.47	.75
* CGI-S_fu	0.09	-0.28, 0.46	.6	0.05	-0.36, 0.45	.83
* CGI-S_post*SSGT	-	-	-	0.22	-0.31, 0.75	.41
* CGI-S_fu*SSGT	-	-	-	0.05	-0.48, 0.58	.85
**Carrier of small CNV (25-100 kb)**						
* CGI-S_post	-1.20	-1.98 -0.43	**.003**	0.01	-0.502, 0.517	.98
* CGI-S_fu	-0.52	-1.38, 0.34	.26	0.03	-0.527, 0.578	.93
* CGI-S_post*SSGT	-	-	-	-1.21	-2.09, -0.33	**.008**
* CGI-S_fu*SSGT	-	-	-	-0.55	-1.52, 0.42	.27
**DD-CGAS (trainer-rated)**	**β**[*]	95%CI	*P*	**β**[*]	95% CI	*P*
Carrier of a rare CNV						
*DD-CGAS_post	-1.32	-4.14, 1.51	.36	0.39	-2.59, 3.36	.80
* DD-CGAS_fu	-1.31	-4.17, 1.54	.37	1.76	-1.31, 4.84	.26
* DD-CGAS_post*SSGT	-	-	-	-1.70	-5.80, 2.41	.42
* DD-CGAS_fu*SSGT	-	-	-	-3.16	-7.36, 1.03	.14
**Carrier of a clinically significant CNV**						
*DD-CGAS_post	-0.20	-4.86, 4.46	.93	2.70	0.66, 4.75	**.0098**
* DD-CGAS_fu	-1.86	-6.70, 2.98	.45	4.01	1.93, 6.08	**.00018**
* DD-CGAS_post*SSGT				-0.20	-7.24, 6.83	.95
* DD-CGAS_fu*SSGT				-2.34	-9.70, 5.03	.53
Carrier of a rare large CNV (>500 kb)						
* DD-CGAS_post	-1.36	-6.03, 3.31	.57	0.82	-6.04, 7.68	.81
* DD-CGAS_fu	-2.45	-7.51, 2.61	.34	4.63	-2.93, 12.19	.23
* DD-CGAS_post*SSGT	-	-	-	-2.20	-10.49, 6.09	.60
* DD-CGAS_fu*SSGT	-	-	-	-7.11	-16.20, 1.97	.13
**Carrier of middle size CNV (101-500 kb)**						
*DD-CGAS_post	-2.09	-5.26, 1.07	.20	-0.17	-3.84, 3.51	.93
*DD-CGAS_fu	-1.15	-4.28, 1.97	.47	1.44	-2.26, 5.13	.45
*DD-CGAS_post*SSGT	-	-	-	-1.82	-6.66, 3.03	.46
*DD-CGAS_fu*SSGT	-	-	-	-2.56	-7.39, 2.28	.30
**Carrier of small CNV (25-100 kb)**						
*DD-CGAS_post	4.27	-2.34, 10.88	.21	0.87	-3.84, 5.57	.72
*DD-CGAS_fu	1.41	-5.90, 8.72	.71	-0.39	-5.50, 4.71	.88
*DD-CGAS_post*SSGT	-	-	-	3.17	-4.94, 11.27	.45
*DD-CGAS_fu*SSGT	-	-	-	1.41	-7.51, 10.33	.76

Abbreviations: ABAS-II the Adaptive Behavior Assessment System II; post, post-intervention; fu, follow-up; DD-CGAS, Developmental Disabilities modification of the Children's Global Assessment Scale; OSU Autism CGI-S, Ohio State University (OSU) Global Severity Scale for Autism; [$]Negative β estimate indicates decrease (symptoms increase) and positive estimate increase (symptoms decrease) of the ABAS-II score from pre-intervention to post-intervention and follow-up for the carriers. [#]Negative β estimate indicates decrease (symptoms decrease) and positive estimate increase (symptoms increase) of the OSU Autism CGI-S score from pre-intervention to post-intervention and follow-up for the carriers. [*]Negative β estimate indicates decrease (symptoms decrease) and positive estimate increase (symptoms increase) of the DD-CGAS score from pre-intervention to post-intervention and follow-up for the carriers.

Table S7. IQ adjusted mixed linear model results for the association between rare genic copy number variation (CNV) and the secondary outcome measures in the active social skills group training (SSGT) participants and the total sample .

	**SSGT KONTAKT group**			**Total sample**		
**ABAS-II (parent-rated)**	**β$**	95%CI	*P*	**β$**	95%CI	*P*
Carrier of a rare CNV						
*ABAS-II_post	10.85	-5.36, 27.06	.19	-6.26	-22.10, 9.58	.44
*ABAS-II_fu	9.21	-7.39, 25.81	.28	4.17	-12.77, 21.11	.63
*ABAS-II_post*SSGT				17.11	-4.59, 38.82	.12
*ABAS-II_fu*SSGT				4.83	-17.92, 27.59	.68
**Carrier of a clinically significant CNV**						
*ABAS-II_post	-22.31	-49.05, 4.42	.10	15.88	5.23, 26.54	**.0037**
*ABAS-II_fu	-14.18	-42.07,13.72	.32	14.29	3.17, 25.42	**.012**
*ABAS-II_post*SSGT				-39.71	-76.57, -2.85	**.035**
*ABAS-II_fu*SSGT				-42.76	-80.43, -5.10	**.027**
**Carrier of a rare large CNV (>500 kb)**						
*ABAS-II_post	-28.22	-56.12, -0.32	.049	12.12	-23.84, 48.08	.51
*ABAS-II_fu	-8.30	-36.26, 19.66	.56	29.16	-6.90, 65.21	.11
*ABAS-II_post*SSGT				-39.98	-84.10, 4.14	.08
*ABAS-II_fu*SSGT				-37.55	-81.78, 6.68	.10
**Carrier of middle size CNV (101-500 kb)**						
*ABAS-II_post	17.84	0.09, 35.59	.05	-7.33	-27.12, 12.45	.47
*ABAS-II_fu	9.58	-8.50, 27.66	.30	-4.56	-24.98, 15.86	0.66
*ABAS-II_post*SSGT				24.97	-0.66, 50.60	.057
*ABAS-II_fu*SSGT				14.04	-12.27, 40.35	.30
**Carrier of small CNV (25-100 kb)**						
*ABAS-II_post	28.46	-9.75, 66.67	.15	-9.29	-34.06, 15.48	0.46
*ABAS-II_fu	22.63	-20.19, 65.46	.30	4.06	-26.60, 34.71	0.80
*ABAS-II_post*SSGT				37.99	-4.91, 80.90	.083
*ABAS-II_fu*SSGT				18.15	-31.67, 67.96	.48
**OSU Autism CGI-S (trainer-rated)**	**β#**	95%CI	*P*	**β#**	95%CI	*P*
**Carrier of a rare CNV**						
*CGI-S_post	-0.01	-0.35, 0.32	.94	0.070	-0.26, 0.40	.67
* CGI-S_fu	-0.11	-0.46, 0.23	.52	-0.049	-0.39, 0.29	.78
* CGI-S_post*SSGT				-0.084	-0.53, 0.37	.72
* CGI-S_fu*SSGT				-0.055	-0.52, 0.41	.82
**Carrier of a clinically significant CNV**						
* CGI-S_post	0.10	-0.45, 0.66	.72	-0.35	-0.58, -0.13	**.0021**
* CGI-S_fu	-0.047	-0.63, 0.53	.87	-0.38	-0.60, -0.15	**.0012**
* CGI-S_post*SSGT				-0.030	-0.80, 0.74	.94
* CGI-S_fu*SSGT				0.14	-0.66, 0.95	.73
**Carrier of a rare large CNV (>500 kb)**						
* CGI-S_post	-0.07	-0.63, 0.49	.80	0.12	-0.63, 0.87	.75
* CGI-S_fu	-0.23	-0.84, 0.37	.45	-0.48	-1.30, 0.35	.26
* CGI-S_post*SSGT				-0.20	-1.11, 0.70	.66
* CGI-S_fu*SSGT				0.23	-0.76, 1.23	.64

**Carrier of middle size CNV (101-500 kb)**						
* CGI-S_post	0.29	-0.09, 0.66	.13	0.07	-0.34, 0.47	.75
* CGI-S_fu	0.09	-0.28, 0.46	.64	0.05	-0.36, 0.45	.82
* CGI-S_post*SSGT				0.22	-0.31, 0.75	.41
* CGI-S_fu*SSGT				0.05	-0.48, 0.58	.85
**Carrier of small CNV (25-100 kb)**						
* CGI-S_post	-1.22	-1.99, -0.44	**.002**	0.08	-0.50, 0.52	.98
* CGI-S_fu	-0.54	-1.40, 0.32	.22	0.02	-0.53, 0.58	.94
* CGI-S_post*SSGT				-1.22	-2.10, -0.34	**.007**
* CGI-S_fu*SSGT				-0.56	-1.53, 0.41	.26
**DD-CGAS (trainer-rated)**	**β***	95%CI	*P*	**β***	95%CI	*P*
Carrier of a rare CNV						
*DD-CGAS_post	-1.28	-4.11, 1.55	.38	0.39	-2.59, 3.37	.80
* DD-CGAS_fu	-1.27	-4.13, 1.59	.39	1.76	-1.31, 4.84	.26
* DD-CGAS_post*SSGT				-1.66	-5.76, 2.44	.43
* DD-CGAS_fu*SSGT				-3.10	-7.29, 1.09	.15
**Carrier of a clinically significant CNV**						
*DD-CGAS_post	-0.14	-4.80, 4.53	.95	2.71	0.67, 4.75	**.0097**
* DD-CGAS_fu	-1.82	-6.67, 3.02	.46	3.99	1.92, 6.06	**.00019**
* DD-CGAS_post*SSGT				-0.16	-7.20, 6.88	.96
* DD-CGAS_fu*SSGT				-2.31	-9.67, 5.05	.54
**Carrier of a rare large CNV (>500 kb)**						
* DD-CGAS_post	-1.33	-6.00, 3.347	.58	0.82	-6.03, 7.68	.81
* DD-CGAS_fu	-2.36	-7.42, 2.71	.36	4.62	-2.94, 12.17	.23
* DD-CGAS_post*SSGT				-2.16	-10.45, 6.12	.61
* DD-CGAS_fu*SSGT				-7.01	-16.09, 2.08	.13
**Carrier of middle size CNV (101-500 kb)**						
*DD-CGAS_post	-2.09	-5.26, 1.08	.20	-0.17	-3.84, 3.51	.93
*DD-CGAS_fu	-1.13	-4.26, 2.00	.48	1.42	-2.27, 5.12	.45
*DD-CGAS_post*SSGT				-1.84	-6.68, 3.01	.44
*DD-CGAS_fu*SSGT				-2.53	-7.36, 2.31	.31
**Carrier of small CNV (25-100 kb)**						
*DD-CGAS_post	4.38	-2.24, 11.00	.20	0.87	-3.83, 5.57	.72
*DD-CGAS_fu	1.55	-5.78, 8.87	.68	-0.35	-5.45, 4.75	.89
*DD-CGAS_post*SSGT				3.39	-4.72, 11.50	.41
*DD-CGAS_fu*SSGT				1.67	-7.25, 10.59	.71

Abbreviations: ABAS-II the Adaptive Behavior Assessment System II; post, post-intervention; fu, follow-up; DD-CGAS, Developmental Disabilities modification of the Children's Global Assessment Scale; OSU Autism CGI-S, Ohio State University (OSU) Global Severity Scale for Autism. $Negative β estimate indicates decrease (symptoms increase) and positive estimate increase (symptoms decrease) of the ABAS-II score from pre-intervention to post-intervention and follow-up for the carriers. #Negative β estimate indicates decrease (symptoms decrease) and positive estimate increase (symptoms increase) of the OSU Autism CGI-S score from pre-intervention to post-intervention and follow-up for the carriers. *Negative β estimate indicates decrease (symptoms decrease) and positive estimate increase (symptoms increase) of the DD-CGAS score from pre-intervention to post-intervention and follow-up for the carriers.
